# Like white on rice: G proteins impact the Hippo pathway to regulate rice grain size

**DOI:** 10.1093/plcell/koaf290

**Published:** 2025-12-26

**Authors:** Renuka Kolli

**Affiliations:** Assistant Features Editor, The Plant Cell, American Society of Plant Biologists; Sainsbury Laboratory, University of Cambridge, Cambridge, United Kingdom

Rice is a staple food of over half of the world's population. With population growth, a 30% increase in global rice demand by 2050 is projected. Rice grain yield is mainly determined by grain weight, grain number per panicle, and panicle number per plant. Several quantitative trait loci for rice grain size have been identified, including several associated with G protein signaling. The heterotrimeric G protein complex, present in all eukaryotes, functions in the perception of environmental and endogenous cues, and influences their growth and development. G protein–coupled receptors typically activate G proteins by promoting the exchange of GDP for GTP in the Gα subunit, triggering its dissociation from the Gβγ heterodimer. GTP-bound Gα and Gβγ then independently interact with downstream effectors for signal transduction. Three Gγ proteins, DEP1, GGC2, and GS3, have been shown to antagonistically regulate rice grain size. DEP1 and GGC2 in complex with Gβ increase grain size, whereas GS3 competitively binds Gβ and decreases grain size (Fig. 1a; Sun et al. 2018). It was also shown that an E3 ligase positively regulates rice grain length by targeting the negative regulator GS3 for degradation (Yang et al. 2021).

**Figure 1.**
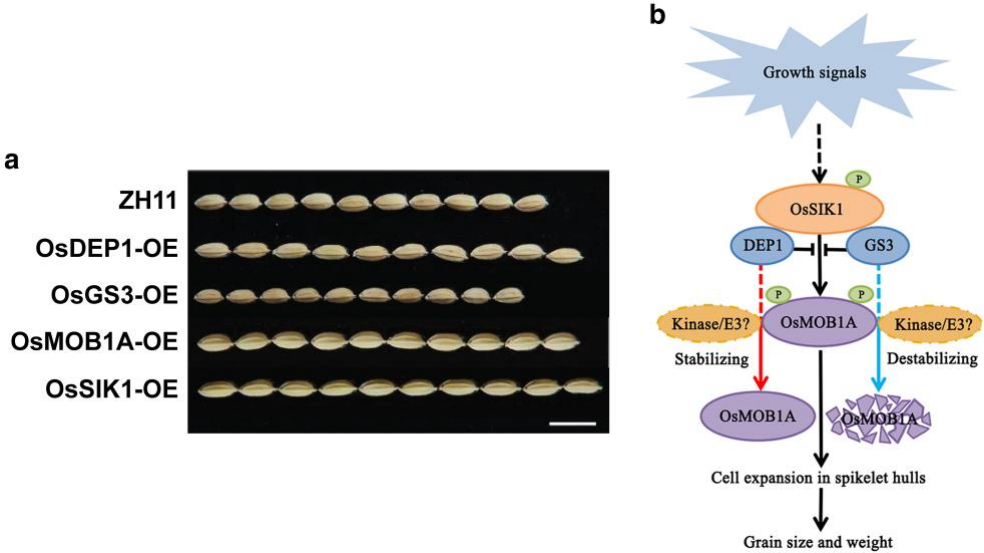
Regulation of rice grain size by G proteins and Hippo pathway proteins. **a)** mature grains of ZH11 and the indicated overexpression (OE) lines. Scale bar = 1 cm. **b)** DEP1 and GS3 interact with SIK1, interfere with the OsSIK1-OsMOB1A interaction, and differentially affect OsMOB1A stability to regulate rice grain size. The differential effects of DEP1 and GS3 on the stability of OsMOB1A are likely mediated by some unknown kinase(s) and/or E3 ligase(s). Adapted from Zhou et al. (2025), supplemental figure 20, figure 6, and figure 8.

**Fangfang Zhou and coauthors (Zhou et al. 2025)** have now uncovered that DEP1 and GS3 regulate rice grain size by modulating the Hippo signaling pathway (Fig. 1b). The Hippo pathway is an evolutionarily conserved signaling network in multicellular organisms that orchestrates cellular proliferation, differentiation, and cell death, which in turn regulate organ size. Dysregulation of the Hippo pathway causes various diseases in humans such as cancer, cardiac diseases, and immune dysfunction. SIK1 and MOB1 are 2 known components of the plant Hippo signaling pathway. Among the 4 OsMOB1 homologs, Zhou et al. decided to focus on OsMOB1A since its sequence is highly similar to the corresponding Arabidopsis homolog, which has been previously shown to play key roles in hormone-mediated plant development (Cui et al. 2016; Guo et al. 2020). They found that *osmob1a* CRISPR mutants produced smaller grains while OsMOB1A-overexpressing lines produced larger grains relative to wild-type ZH11 control grains (Fig. 1a). Similarly, the grains of os*sik1* CRISPR mutants were smaller while those of OsSIK1-overexpressing lines were larger than ZH11 grains (Fig. 1a). Hence, both OsMOB1A and OsSIK1 positively regulate grain size in rice.

Increased cell size but similar cell number in the outer epidermis of mature grains of both the over expression lines indicated that OsMOB1A and OsSIK1 regulate rice grain size by promoting cell elongation in spikelet hulls. Using complementary protein-protein interaction assays, the authors demonstrated a direct interaction between OsSIK1 and OsMOB1A. In vitro and in vivo kinase assays revealed that OsSIK1 auto-phosphorylates and phosphorylates OsMOB1A. It was found that the latter enhances the stability of OsMOB1A because OsMOB1A protein levels were increased in OsSIK1-overexpressing lines but decreased in *ossik1* mutants relative to ZH11. As further confirmation, a phosphomimic form of OsMOB1A remained stable while a nonphosphorylated mutant form degraded faster than OsMOB1A in a cell-free protein degradation assay.

By yeast 2-hybrid screening using known regulators, the authors identified that DEP1 and GS3 interact with OsSIK1 and confirmed the interactions with in vitro pull-down assays. After finding that OsSIK1 did not phosphorylate DEP1 and GS3, they investigated the phosphorylation of OsMOB1A by OsSIK1 in the presence of DEP1 or GS3. In both in vitro and in vivo assays, the phosphorylation levels of OsMOB1A by OsSIK1 were decreased in the presence of DEP1 or GS3 in a dose-dependent manner. Moreover, the OsSIK1-OsMOB1A interaction was weakened by DEP1 or GS3. In agreement with the known antagonistic grain size regulation by DEP1 and GS3 (Sun et al. 2018), the in vivo stability of OsMOB1A was found to negatively correlate with GS3 and positively with DEP1. Genetic crossing experiments indicated that these Gγ proteins act upstream of the Hippo pathway.

Thus, Zhou et al. (2025)found that the G protein signaling pathway acts upstream of the Hippo signaling to regulate rice grain size and weight (Fig. 1b). Their findings provide potential targets for improving rice yield. Future research to better understand the Hippo signaling pathway and its upstream regulators in plants would answer fundamental questions about plant organ development and identify more candidates for improving crop yields for global food security. It may also help in developing novel therapies to cure human diseases associated with alterations in the Hippo pathway.

## Recent related articles in *The Plant Cell*:

Li et al. (2025) elucidated a DEP1-mediated signaling pathway linking G-proteins to brassinosteroid signaling.

Sun et al. (2025) found that the tails of the Gγ proteins DEP1, GGC2, and GS3 determine their distinct functions and downstream signal transduction.

Chen et al. (2024) revealed a pathway involving OsMOB1A and a cyclin-dependent kinase module that regulates rice grain size and weight.

## Dive Curated Terms

The following phenotypic, genotypic, and functional terms are of significance to the work described in this paper:

DEP1 Gramene: Os09g26999
DEP1 Araport: Os09g26999

GGC2 Gramene: Os08g0456600
GGC2 Araport: Os08g0456600

GS3 Gramene: Os03g0407400
GS3 Araport: Os03g0407400

MOB1A Gramene: Os03g38020
MOB1A Araport: Os03g38020

SIK1 Gramene: Os03g54780
SIK1 Araport: Os03g54780
